# Carbon Nanowalls as Anode Materials with Improved Performance Using Carbon Nanofibers

**DOI:** 10.3390/nano13192622

**Published:** 2023-09-22

**Authors:** Kangmin Kim, Chris Yeajoon Bon, Junghyun Kim, Jang Myoun Ko, Wonseok Choi

**Affiliations:** 1 Department of Electrical Engineering, Hanbat National University, Daejeon 34158, Republic of Korea; talk9797@naver.com; 2 Department of Materials Science and Engineering, Research Institute of Advanced Materials (RIAM), Seoul National University, Seoul 08826, Republic of Korea; chrisbon@snu.ac.kr; 3 Department of Advanced Materials Engineering, Hanbat National University, Daejeon 34158, Republic of Korea; jhkim2011@hanbat.ac.kr; 4 Department of Chemical and Biological Engineering, Hanbat National University, Daejeon 34158, Republic of Korea; jmko@hanbat.ac.kr

**Keywords:** carbon nanowalls, carbon nanofiber, PAN, electrospinning method, lithium-ion batteries

## Abstract

In this paper, a new synthesis of carbon nanofibers (CNFs)/carbon nanowalls (CNWs) was performed to improve the characteristics of anode materials of lithium-ion batteries by using the advantages offered by CNWs and CNFs. Among the carbon-based nanomaterials, CNWs provide low resistance and high specific surface area. CNFs have the advantage of being stretchable and durable. The CNWs were grown using a microwave plasma-enhanced chemical vapor deposition (PECVD) system with a mixture of methane (CH_4_) and hydrogen (H_2_) gases. Polyacrylonitrile (PAN) and N,N-Dimethyl Formamide (DMF) were stirred to prepare a solution and then nanofibers were fabricated using an electrospinning method. Heat treatment in air was then performed using a hot plate for stabilization. In addition, heat treatment was performed at 800 °C for 2 h using rapid thermal annealing (RTA) to produce CNFs. A field emission scanning electron microscope (FE-SEM) was used to confirm surface and cross-sectional images of the CNFs/CNWs anode materials. Raman spectroscopy was used to examine structural characteristics and defects. Cyclic voltammetry (CV), electrochemical impedance spectroscopy (EIS), and constant current charge/discharge tests were performed to analyze the electrical characteristics. The synthesized CNFs/CNWs anode material had a CV value in which oxidation and reduction reactions were easily performed, and a low Rct value of 93 Ω was confirmed.

## 1. Introduction

Lithium-ion batteries, which are widely used energy storage devices, have many characteristics including fast charging and discharging, high energy density, and practicality [1,2]. Lithium-ion batteries are one of the essential elements for commercial products including future electric vehicles, household items, and smart grids. A lithium-ion battery is largely composed of an anode, a cathode, a separator, and an electrolyte. There is a high demand for carbon materials, nickel, and tin oxide as they are mainly used as anode materials for lithium-ion batteries [3,4]. However, the theoretical capacity of the carbon anode material is relatively low at 372 mAhg^−1^, and the stability of nickel and tin oxide is not guaranteed. Therefore, among carbon-based nanomaterials, carbon, carbon nanotube (CNT), and hard carbon have been used as anode materials for lithium-ion batteries through many studies [5,6,7,8,9]. Carbon-based nanomaterials have stable durability and low resistance, so lithium-ion electrons move quickly [10,11,12]. In this study, carbon nanowalls (CNWs), one of the carbon allotropes, were synthesized with carbon nanofibers (CNFs) to fabricate a new structured carbon anode material using carbon-based nanomaterials. CNWs are composed of vertical growth structures, and are considered suitable for use as anode materials due to their large specific surface area and low resistance [13,14,15]. However, if CNWs are used alone as an anode material, the vertically grown carbon walls of CNWs can collapse due to electrochemical reactions that occur during the charging and discharging process of lithium-ion batteries. Consequently, we synthesized CNFs on CNWs to compensate for this problem. Through a heat treatment process, the cross-linked structure of polyacrylonitrile (PAN) nanofibers is broken, and then, through a carbonization process, CNFs become more stretchable [16,17,18]. This new technique proposes a new composite structure of CNFs/CNWs that can use the advantages of CNWs, such as a large specific surface area and low resistivity, by synthesizing CNFs on top of CNWs.

## 2. Materials and Methods

### 2.1. Preparation of Substrate

TiN is a typical interlayer with excellent thermal stability and chemical safety, and with high fine hardness [19]. Also, in our previous study, the TiN interlayer was relatively excellent among battery characteristics using Ti, Cr, and TiN as an interlayer between copper foil and CNWs [20]. Therefore, an interlayer was deposited to improve the adhesion between copper foil and CNWs, which are used as current collectors. Using an RF magnetron sputtering system, titanium nitride (TiN) was used as an interlayer on copper foil that had been ultrasonically cleaned for 10 min in the following order: trichlorethylene (TCE) (≥99.5%), acetone (≥99.5%), methanol (≥99.8%), and deionized water (DI water) (≥18 MΩ∙cm, 25 °C).

### 2.2. Growth of CNW

TiN-deposited copper foil was placed on a stage inside a chamber of a microwave plasma-enhanced chemical vapor deposition (PECVD) system (ASTeX-type, Woosin CryoVac, Republic of Korea, Uiwang), and a vacuum of 10^−5^ Torr was maintained. To form plasma, 20 sccm of methane gas and hydrogen gas were injected at a ratio of 2:1, and the microwave power was set to 1200 W. The walking pressure was maintained at 4 × 10^−2^ Torr for the growth of CNWs after reaching the base pressure. The substrate was maintained at 600 °C to grow CNWs for 20 min. After this, it was slowly cooled by maintaining a low vacuum and was taken out of the chamber.

### 2.3. Electrospinning System

Before fabricating nanofibers, a solution required for an electrospinning system (electrospinning machine, IDK lab, Korea) was prepared. For this purpose, 1 g of Polyacrylonitrile (PAN) and 9 g of N,N-Dimethylformamide (DMF) were stirred for 16 h to obtain a completely dissolved 10 wt% solution. The solution was put into a syringe with an 18G needle. A syringe volume of 10 mL was set at an injection rate of 13 uL/min with an injection volume of 5 mL. Radiation was performed at 13.38 kV power. At this time, the distance was maintained after separating the electrospinning needle and the CNWs sample.

### 2.4. Fabrication of Carbon Nanofiber

**Figure 1:**
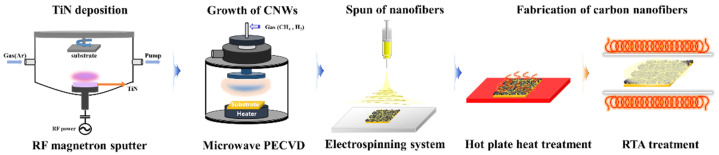
A schematic diagram of the material preparation process.

The spun nanofibers were heat-treated using rapid thermal annealing (RTA) and a hot plate to convert them into CNFs. The heat treatment process is divided into air stabilization and carbonization. For the air stabilization, the nanofibers were heated on an aluminum plate on top of a hot plate at 270 °C for 2 h in air. After stabilization, a carbonization process was performed at 800 °C for 2 h in a vacuum atmosphere of 10^−2^ Torr using an RTA chamber. Figure 1 is a schematic diagram of the overall fabrication process for the structure of CNFs/CNWs. We conducted experiments in the following sequence: RF magnetron sputtering, microwave PECVD, electrospinning system, heat treatment, and RTA heat treatment.

### 2.5. Production of Lithium-Ion Battery

A coin cell (half-cell) was fabricated to confirm the characteristics of the anode material of a lithium-ion battery. Prior to manufacturing the battery, the anode material was assembled through a punching process. The coin cell consists of 5 parts (2030, 1.0t, Wlcos, Republic of Korea, Gunpo). For the electrolyte, 1M lithium hexafluorophosphate (LiPF6, Panax Etec) solvent with EC/DMC = 1/1 (*v*/*v*) was used. Metal lithium (150 μm) was used as a counter electrode while polyethylene (Celgard, 20 μm) was used as a separator and assembled in an argon-filled glove box. We added approximately 25 mL of electrolyte to the coin cell. Subsequently, CNWs were grown at a rate of 6 mg/h to reach a mass of 0.002 g, and CNFs, produced through a 2 h carbonization process, were fabricated with a mass of 0.008 g at a rate of 8 mg/h. The ratio of CNFs to CNWs in the anode material was 1:4.

### 2.6. Characterization and Analysis of the Samples

CNWs and CNFs were fabricated under the same conditions to compare the anode material properties of the CNFs/CNWs. Surface and cross-sectional images of the CNWs and CNFs/CNWs were obtained from a field emission scanning electron microscope (FE-SEM; S-4800, HITACH, Tokyo, Japan), while surface images of the CNFs were obtained from the FE-SEM. The morphology of the CNFs/CNWs was characterized using a transmission electron microscope (FE-TEM;HF-500, HITACH, Japan). Raman spectroscopy (LabRAM HR-800, HORIBA, Japan, Kyoto) was performed to observe the structural characteristics of each sample. The Raman spectrum provided information such as the unique graphene layer of carbon materials, peaks, and defects. The electrochemical characteristics of coin cells using anode material samples were analyzed. A PEBC050.1 battery cycler (PEBC050, PNE SOLUTION, Suwon, Korea) was used for cycling at various current densities in the potential range of 0.01 V to 2 V. The charge rate performance of the secondary battery was tested by charging and discharging at the same current densities of 0.5, 1, and 2 Ag^−1^ for 5 cycles, respectively. A cycle life test was then performed, in which the cell was charged and discharged at 1 Ag^−1^ for 1000 cycles. In addition, cyclic voltammetry (CV) was performed at scan rates of 2 mVs^−1^ and 10 mVs^−1^ in the potential range of 0.01 V to 2 V. Electrochemical impedance spectroscopy (EIS) was performed in the frequency range of 10 mV, 10 mHz to 100 kHz, using an AUTOLAB instrument (PGSTAT100, Eco Chemie, Utrecht, The Netherlands).

## 3. Results and Discussion

### 3.1. Structural Characteristics of the Samples

**Figure 2:**
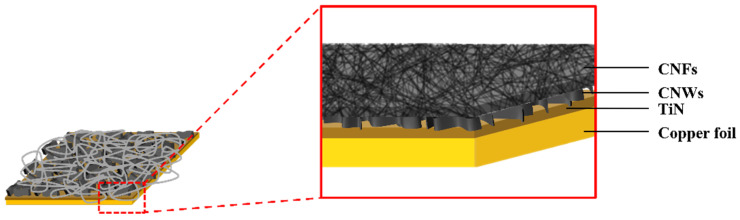
A schematic diagram of the CNFs/CNWs structure.

**Figure 3:**
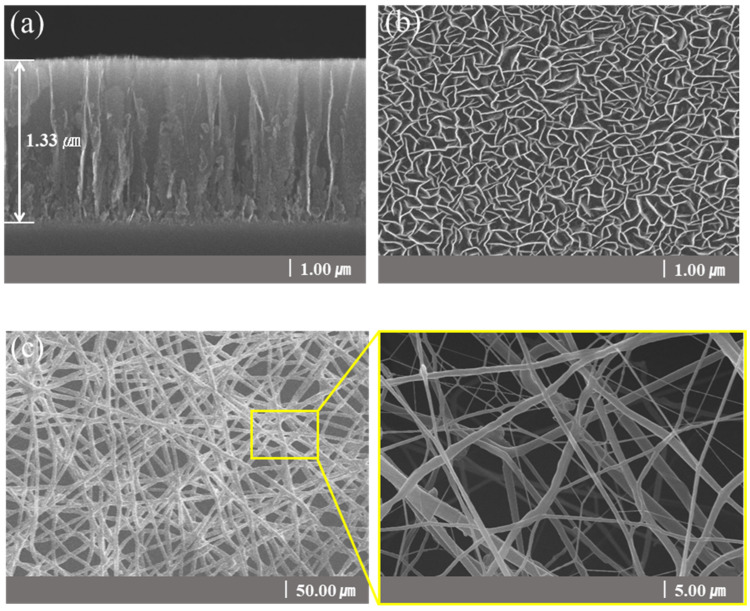

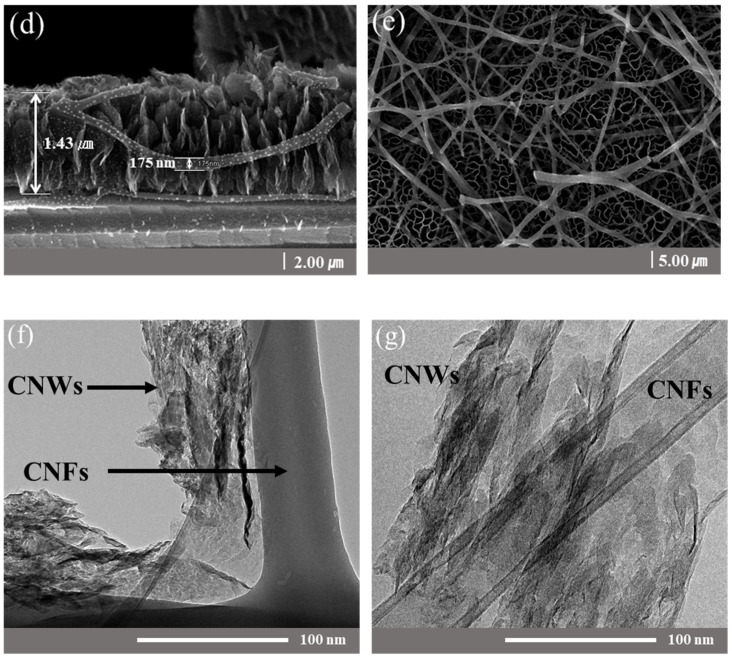
FE-SEM images of a sample synthesized on a Si wafer. Cross-sectional images: (**a**) CNWs, (**d**) CNFs/CNWs. Surface images: (**b**) CNWs, (**c**) CNFs, and (**e**) CNFs/CNWs. FE-TEM images of CNFs/CNWs structure: (**f**,**g**).

Figure 2 is a structural schematic diagram of CNFs/CNWs fabricated as anode materials. An TiN interlayer was deposited on copper foil, CNWs were grown with PEVCD, and CNFs were fabricated through a carbonization process. Figure 3 shows surface and cross-sectional images of samples synthesized with CNWs, CNFs, and CNFs/CNWs on a Si substrate in the same way as the anode materials were fabricated for FE-SEM and Raman analysis. Figure 3a,b are cross-section and surface images of CNWs; the thickness was about 1.33 μm. Figure 3c,d are images of CNFs carbonized using the PAN solution. They were entangled in a spider web-like structure, and there was almost no beads phenomenon. Figure 3e,f show cross-sectional and surface images of the CNFs/CNWs structure in which CNFs were synthesized on top of CNWs. CNFs have a thickness of about 175 nm, and CNWs are maintained even during the process of making CNFs. From the structures in Figure 3d,e, the stable synthesis of CNWs and CNFs can be confirmed. Figure 3f,g are TEM images of the synthesized CNFs/CNWs; through these images, it can be confirmed that a multilayer graphene structure of CNWs is combined with CNFs.

### 3.2. Raman Spectra of the Anode Material Samples

**Figure 4:**
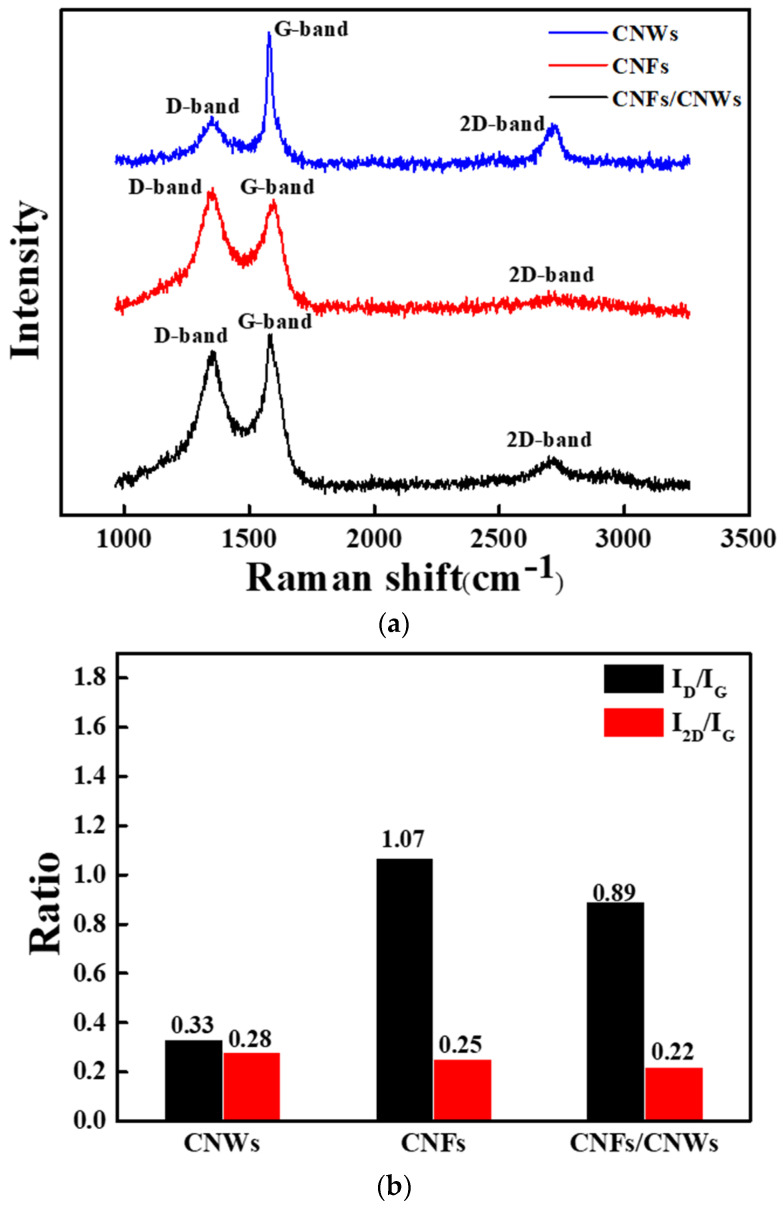
Raman analysis for each carbon anode material. (**a**) Raman spectra, (**b**) I_D_/I_G_, I_2D_/I_G_ ratio.

Figure 4 shows the results of Raman analysis of carbon anode materials using three materials. In Figure 4a, D, G, and 2D bands appearing in the carbon series were observed [21,22,23,24]. Among the three anode materials, CNWs showed a relatively high G peak, which is commonly seen in carbon-based materials, and was confirmed at 1580 cm^−1^. The D peak, which represents the defect structure of graphite and amorphous carbon system, was observed at 1352 cm^−1^. The 2D peak observed at 2714 cm^−1^ represents a π-π bond and shows a slightly higher value compared to the previous two, as a high intensity peak appears when the number of graphene layers is relatively small. CNFs showed a D peak at 1349 cm^−1^ and a G peak at 1598 cm^−1^. CNFs have a fibrous structure, and a 2D peak was confirmed at 2754 cm^−1^, but intensity was almost absent. CNFs/CNWs showed D, G and 2D peaks at 1349 cm^−1^, 1586 cm^−1^, and 2714 cm^−1^, respectively. The 2D peak, which was almost invisible in CNFs, appeared clearly after synthesizing with CNWs, while the D peak was relatively reduced. A 2D peak was observed since it was synthesized with CNWs affected by π-π bonds, while the D peak, which is a defective structure, seems to be reduced [25,26,27]. The ratios of I_D_/I_G_ and I_2D_/I_G_ can be confirmed in Figure 4b. The comparatively small I_2D_/I_G_ values of CNFs/CNWs indicate that they are structured with a number of thick graphene layers [28,29]. In addition, structural stability is improved by synthesizing CNWs with low I_D_/I_G_ values and CNFs with high I_D_/I_G_ values.

### 3.3. Cyclic Voltammetry (CV) of the Anode Material Samples

**Figure 5:**
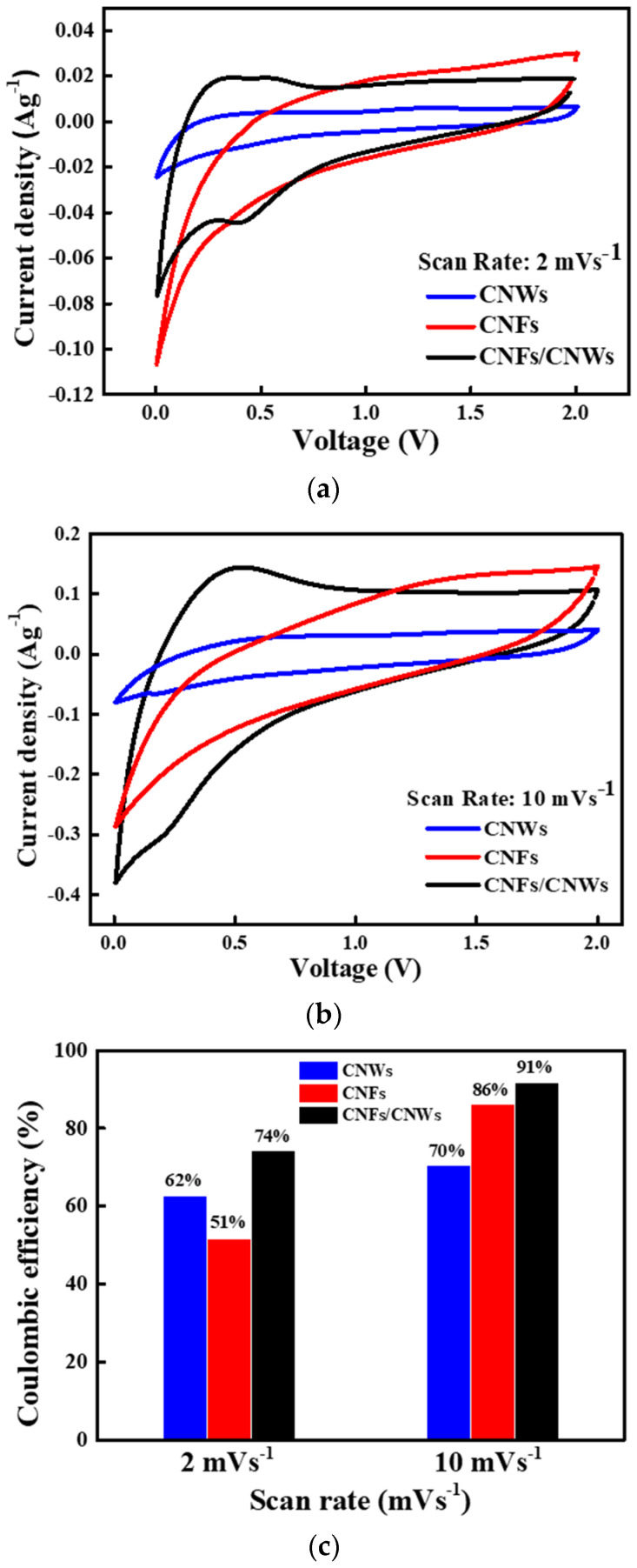
CV measurement for each carbon anode material. (**a**) CV at current density of 2 mVs^−1^, (**b**) CV at current density of 10 mVs^−1^, and (**c**) Coulombic efficiency (%) by current density.

Figure 5 shows the result of measuring cyclic voltammetry (CV) by fabricating a half cell using each carbon anode material. In Figure 5a, CVs can be confirmed by applying 2 mVs^−1^ to cells using CNWs anode materials, CNFs anode materials, and CNFs/CNWs anode materials. A low reduction potential and oxidation potential can be confirmed after the first CV cycle of the cell using CNWs anode materials. On the other hand, a high reduction potential and oxidation potential can be confirmed in the CV of the cell using the CNFs anode materials. Consequently, if the two are synthesized, an improved CV can be expected. The CV of the CNFs/CNWs anode materials compensates for the disadvantages of (a) and (b). After the first cycle, the reduction potential was maintained, and the oxidation potential was low and fast. The CV was then measured at 10 mVs^−1^ by increasing the current density of (b). The current density was improved compared to the result measured at 2 mVs^−1^, which was due to the synthesis of CNWs and CNFs, while durability was more robust. Figure 5c shows the result of Coulombic efficiency (%) of carbon anode materials by current density. When using the anode material synthesized with CNWs and CNFs, it shows better values at 74% and 91%. The advantages of the large specific surface area of CNWs and the durability of CNFs have increased the cycle life of the anode materials.

### 3.4. Electrochemical Impedance Spectroscopy (EIS) of the Anode Material Samples

**Figure 6:**
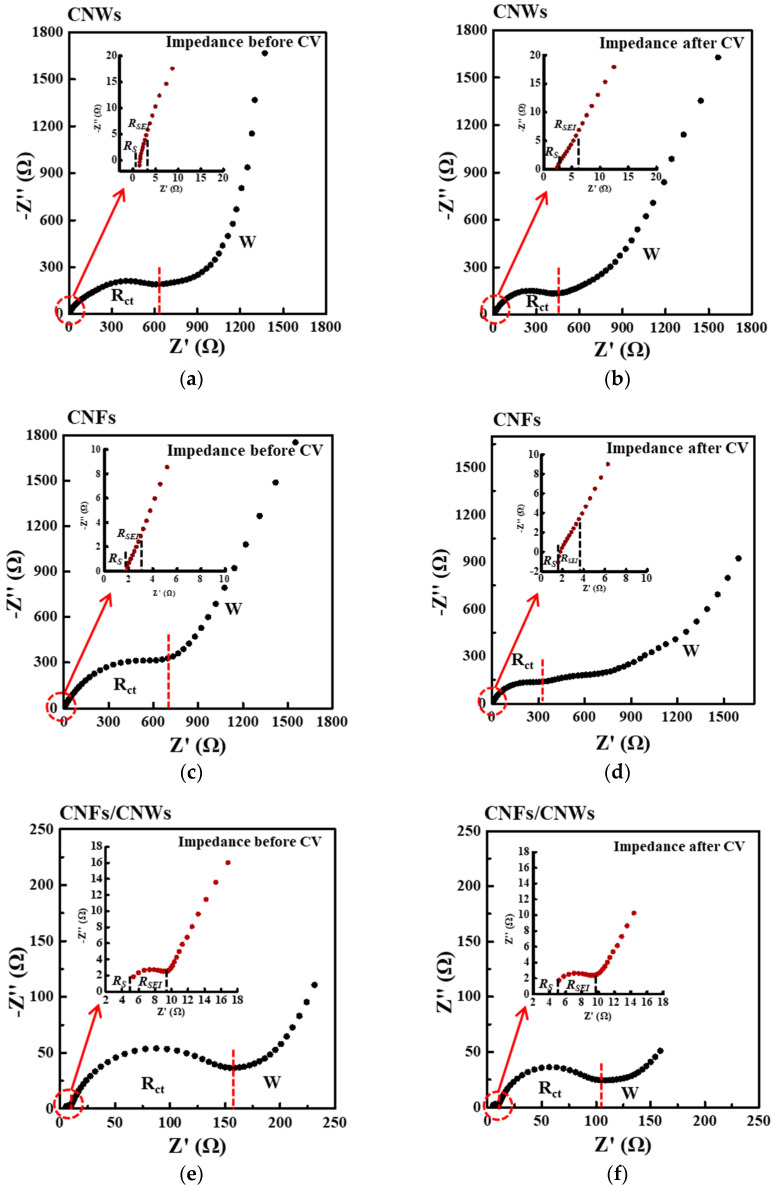
The effect of SEI formation on electrode resistance characterized by electrochemical impedance spectroscopy (EIS). (**a**,**b**) CNWs, (**c**,**d**) CNFs, and (**e**,**f**) CNFs/CNWs.

Figure 6 shows the electrochemical impedance spectroscopy (EIS) results of each cell for each carbon anode material. EIS was measured before and after CV measurement to confirm changes in R_s_, R_SEI_, R_ct_, and Warburg impedance. First, for the change in the cell using the CNW anode material, R_s_ increased from 1.42 Ω to 2.40 Ω, and R_SEI_ decreased from 1.02 Ω to 1.09 Ω. R_ct_ also decreased from 571 Ω to 436 Ω, and the slope of Warburg impedance decreased. For the change in the cell using the CNFs anode material, R_s_ decreased slightly from 1.78 Ω to 1.73 Ω, while R_SEI_ decreased from 1.02 Ω to 0.58 Ω. R_ct_ also decreased significantly from 676 Ω to 310 Ω, and the slope of Warburg impedance decreased. For the change in the cell using the CNFs/CNWs anode materials, R_s_ decreased slightly from 5.37 Ω to 5.17 Ω, while R_SEI_ increased from 4.13 Ω to 4.46 Ω. R_ct_ also decreased from 155 Ω to 93 Ω, and the slope of Warburg impedance slightly increased. The new structure of CNFs/CNWs anode materials exhibits a fast movement of ion electrons and stability with low resistance Table 1.

## 4. Conclusions

In this paper, a new structure of CNFs/CNWs anode material using CNFs was synthesized to improve the performance of secondary battery anode materials, and characteristic analysis was subsequently performed. CNWs were grown with a microwave PECVD system on the current collector of a lithium-ion battery, and nanofibers were spun using an electrospinning system. The nanofibers were then stabilized in air on a hot plate, and the carbonization process was performed with high-temperature heat inside the RTA chamber. In the CV measurement, CNFs/CNWs showed better current density than CNWs and CNFs, and the Coulombic efficiency (%) also showed relatively high values at 74% and 91%. In EIS, the R_SEI_ value was as low as 4.45 Ω, and the R_ct_ value was 93 Ω, yielding a low resistance value. The new structure, CNFs/CNWs anode material, was synthesized with CNFs to complement the advantages of CNWs’ wide specific surface area and low resistance, as well as the stability of the vertical growth structure. In addition, the fast mobility of lithium-ion electrons and better charge/discharge efficiency were confirmed with low electrode resistance and improved Coulombic efficiency. CNFs/CNWs anode materials have sufficient potential to complement high specific capacity and structural safety through further CNFs/CNWs research, and have shown the unlimited development potential of various carbon-based nanomaterials as anode materials.

## Figures and Tables

**Table 1:** R_s_, R_SEI_, and R_ct_ values before and after CV measurement.

**Before the CV (** **Ω)**	**CNWs**	**CNFs**	**CNFs/CNWs**
R_s_	1.42	1.78	5.37
R_SEI_	1.02	1.02	4.13
R_ct_	571	676	155
**After the CV (** **Ω)**	**CNWs**	**CNFs**	**CNFs/CNWs**
R_s_	2.40	1.73	5.17
R_SEI_	1.09	0.58	4.45
R_ct_	436	310	93

## Data Availability

The data presented in this study are available in the article.
